# The small-molecule kinase inhibitor D11 counteracts 17-AAG-mediated up-regulation of HSP70 in brain cancer cells

**DOI:** 10.1371/journal.pone.0177706

**Published:** 2017-05-18

**Authors:** Susanne Schaefer, Tina H. Svenstrup, Barbara Guerra

**Affiliations:** Department of Biochemistry and Molecular Biology, University of Southern Denmark, Odense, Denmark; University of Kansas Medical Center, UNITED STATES

## Abstract

Many types of cancer express high levels of heat shock proteins (HSPs) that are molecular chaperones regulating protein folding and stability ensuring protection of cells from potentially lethal stress. HSPs in cancer cells promote survival, growth and spreading even in situations of growth factors deprivation by associating with oncogenic proteins responsible for cell transformation. Hence, it is not surprising that the identification of potent inhibitors of HSPs, notably HSP90, has been the primary research focus, in recent years. Exposure of cancer cells to HSP90 inhibitors, including 17-AAG, has been shown to cause resistance to chemotherapeutic treatment mostly attributable to induction of the heat shock response and increased cellular levels of pro-survival chaperones. In this study, we show that treatment of glioblastoma cells with 17-AAG leads to HSP90 inhibition indicated by loss of stability of the EGFR client protein, and significant increase in HSP70 expression. Conversely, co-treatment with the small-molecule kinase inhibitor D11 leads to suppression of the heat shock response and inhibition of HSF1 transcriptional activity. Beside HSP70, Western blot and differential mRNA expression analysis reveal that combination treatment causes strong down-regulation of the small chaperone protein HSP27. Finally, we demonstrate that incubation of cells with both agents leads to enhanced cytotoxicity and significantly high levels of LC3-II suggesting autophagy induction. Taken together, results reported here support the notion that including D11 in future treatment regimens based on HSP90 inhibition can potentially overcome acquired resistance induced by the heat shock response in brain cancer cells.

## Introduction

Glioblastoma is the most common and aggressive type of primary brain tumor in adults associated with a poor prognosis and, in general, a modest response to all treatment modalities. Because of its lethalness, glioblastoma has been the first type of malignant tumor that has been sequenced as part of The Cancer Genome Atlas (TCGA) pilot study [1]. A systematic examination of the glioblastoma genome revealed a list of molecular alterations which may explain the ability of this type of tumor to adapt in response to target therapy [1,2]. Interestingly, a large number of activated oncoproteins is dependent on the expression of functional heat shock protein 90 (HSP90) in complex with CDC37 and contributes to an increase in survival, growth and resistance to treatment of cancer cells [3,4]. Because of the broad spectrum of proteins dependent on intact chaperone activity, HSP90 has become an attractive therapeutic target for cancer treatment.

17-(Allylamino)-17-demethoxygeldanamycin (17-AAG), an analog of geldanamycin, is among the HSP90 inhibitors that has been shown to promote growth inhibition in a number of cancer cell lines as well as anti-tumor activity in clinical trials [5,6]. Interestingly, although HSP90 is well expressed in the majority of normal and cancer cells, the binding affinity of 17-AAG to HSP90 is 100-fold higher in tumor cells than in normal cells enabling selective targeting of this protein in cancer cells [7]. 17-AAG and its analogues have attracted major interest for the therapeutic targeting of glioblastoma because of the high lipophilicity, which would enable it to across the blood-brain barrier. However, *in vitro* and *in vivo* studies conducted with HSP90 inhibitors have not always provided promising results because of the presence of redundant signaling pathways and/or molecular changes occurring in response to prolonged treatment [8].

Several studies have shown that acquired resistance to 17-AAG treatment may derive from induction of anti-apoptotic HSP70 and members of its family (e.g. HSC70) as an off-target effect of HSP90 inhibition [9,10]. Indeed, studies aiming at reducing the expression of HSC70 and HSP70 simultaneously in combination with HSP90 inhibition showed a remarkable increase in toxicity and cell death suggesting that a combined treatment could prove to be effective in the management of various types of cancer including glioblastoma [11,12].

We have recently reported evidence that inhibition of protein kinase CK2 leads to down-regulation of HSP70 in hepatoma cells treated with the proteasome inhibitor MG132 [13]. CK2 is a Ser/Thr tetrameric protein kinase composed of two catalytic α and α’-subunits and two regulatory β-subunits involved in a wide variety of cellular processes (for reviews see [14–16]). As a consequence of its pro-survival and anti-apoptotic functions, CK2 has become a valuable target in cancer therapy, in recent years.

In view of the potential therapeutic benefits resulting from simultaneous inhibition/down-regulation of HSP70 and HSP90 in cancer cells [17], we asked the question whether combined inhibition of HSP90 and CK2 resulted in enhanced cytotoxicity in glioblastoma cells. Indeed, our data show this is the case and suggest that this strategy could provide a new aspect for therapeutic intervention in the management of brain cancer cells with acquired resistance to HSP90 inhibitors.

## Materials and methods

### Cell culture and treatment

U-87 MG and M059K cell lines were obtained from the American Type Culture Collection (ATCC, Rockville, MD, USA) and cultivated in Dulbecco’s modified Eagle’s medium (DMEM, Invitrogen, Taastrup, Denmark) supplemented with 10% fetal bovine serum (FBS, Biochrom AG, Berlin, Germany) at 37°C under a 5% CO_2_ atmosphere. Cells were treated with 17-(Allylamino)-17-demethoxygeldanamycin (17-AAG, Sigma-Aldrich, Schnelldorf, Germany), 1,3-Dichloro-6-[(E)-((4-methoxyphenyl)imino)methyl] diben- zo(b,d) furan-2,7-diol, referred to as D11 (DTP, NIH/NCI, Rockville, MD, USA), MG132 (Sigma-Aldrich), CX-4945 (Selleck Chemicals, Houston, TX, USA), 4-[(E)-(fluoren-9-ylidenehydrazinylidene)-methyl] benzoic acid (referred to as E9, DTP, NIH/NCI, USA), Glutamine (Gln, Sigma-Aldrich) and Bafilomycin A1 (Sigma-Aldrich) as indicated in the figure legends. Cells were cultured in Gln-free medium for 12 h prior incubation with 2 mM Gln for 12 h. Down-regulation of protein kinase CK2 and HSP70 was carried out by small interfering RNA (siRNA, Dharmacon, CO, USA) essentially as described in [18]. The WST-1 assay (Roche, Hvidovre, Denmark) was performed to measure cell viability determined in a microtiter plate reader according to the manufacturer’s recommendations (Perkin-Elmer, Waltham, MA, USA).

### Preparation of whole cell lysate, western blot analysis and antibodies

Cells for Western blot analysis were harvested and whole protein extracts were obtained following a protocol described before [19,20]. The following primary antibodies were employed in the study: rabbit monoclonal anti-NF-κB/RelA, rabbit monoclonal anti-phospho-AKT(S473), mouse monoclonal anti-phospho-p70S6K(T389), mouse monoclonal anti-HSP27, rabbit monoclonal anti-HSF1, rabbit monoclonal anti-LC3A and rabbit polyclonal anti-HSP70 (all from Cell Signaling Technology, Beverly, MA, USA); mouse monoclonal anti-β-actin (Sigma-Aldrich); rabbit polyclonal anti-phospho-NF-κB/p65 [(S529), Abcam, Cambridge, MA, USA]; rabbit polyclonal anti-EGFR, rabbit polyclonal anti-p70S6K and rabbit polyclonal anti-HSP90 (all from Santa Cruz Biotechnology); rabbit polyclonal anti-AKT1 (Merck-Millipore, Billerica, MA, USA); rabbit monoclonal anti-phospho-HSF1 [(S326), GeneTex, Wembley, UK]. Rabbit polyclonal anti-CK2α’ was obtained by immunizing rabbits with a specific peptide sequence of human CK2α’ (SQPCADNAVLSSGTAAR). Rabbit polyclonal anti-CK2α was obtained by immunizing rabbits against the human full-length protein sequence.

### Luciferase reporter assay

The transcription activity of HSF1 was determined by transfecting cells with a luciferase reporter vector (pHSE-Luc) bearing the cis-acting enhancer element sequence which drives expression of the firefly luciferase reporter gene upon HSF1 binding (Panomics-Affymetrix, Santa Clara, CA, USA). Control experiments were performed transfecting cells with an empty vector. Transfection of cells was carried out with Lipofectamine 3000 (Invitrogen). After 24 h from transfection, cells were incubated with 0.5 μM 17-AAG and 50 μM D11 for additional 24 h, respectively. Where indicated, cells were treated with 50 μM MG132 in the last 12 h of incubation time or grown at 43°C for 1 h followed by 23 h at 37°C. Quantitation was performed applying the Luciferase assay system following the manufacturer’s guidelines (Promega, Stockholm, Sweden).

### Immunostaining

Immunostaining was carried out essentially as described in [21] employing rabbit monoclonal anti-HSF1 antibody (Cell Signaling Technology) followed by incubation with biotinylated swine anti-rabbit immunoglobulin (Dako, Glostrup, Denmark) and streptavidin-conjugated Alexa Fluor 488 (Thermo Fisher Scientific, Waltham, MA, USA). Cells were counterstained with 4’,6’-diamidino-2-phenylindole (DAPI, Sigma-Aldrich) and analyzed on a Leica DMRBE microscope equipped with a DFC 420C camera and Leica Application Suite V 3.3.0 software (Leica Microsystem, Wetzler, Germany) at 400x magnification.

### Gene expression profiling

Total RNA samples preparation was carried out by phenol-chloroform extraction and subsequent silica-membrane-based purification in combination with on-column DNAse digestion with the miRNeasy kit (Qiagen, Hilden, Germany) following the manufacturer’s instructions. The cDNA was prepared using the RT^2^ First strand kit (Qiagen). The expression analysis of 84 heat shock protein genes was analyzed with the Qiagen RT^2^ Profiler PCR array according to the manufacturer’s guidelines in 96-well plates with a StepOnePlus^TM^ real-time cycler (Applied Biosystems, Nærum, Denmark). Data were normalized to five housekeeping genes included in the kit. Normalized data were analyzed using the ΔΔC_T_ method with the equation ΔΔC_T_ = ΔC_T_ (experimental sample group)– ΔC_T_ (control group), and the fold-change was calculated based on ΔΔC_T_ with 2^-ΔΔCT^ for positive changes or with -1/2^-ΔΔCT^ for negative changes [22,23].

### Statistical analysis

The experiments were performed a number of times as indicated in the figure legends. Data are presented as means +/- STDEV. Statistically significant differences were calculated by the two-tailed *t*-test (student’s *t*-test). The levels of significance are indicated in the figure legends.

## Results

### Destabilization of HSP70 in glioblastoma cells is mediated by D11 and partially dependent on CK2 inhibition or down-regulation

**Fig 1:**
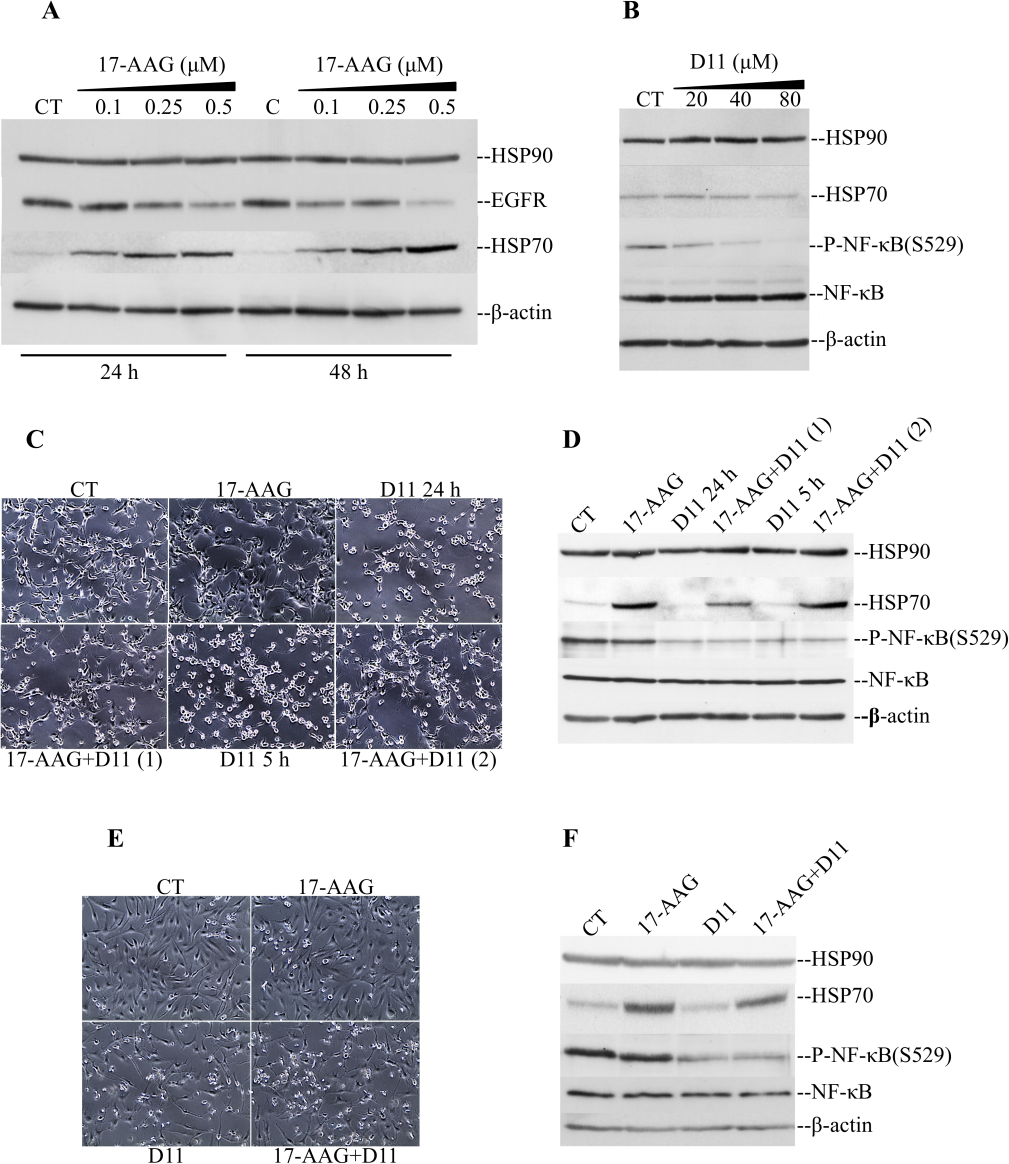
Effect of 17-AAG on the expression of HSP70 in glioblastoma cells. (A) U-87 MG cells were treated with increasing concentrations of 17-AAG for 24 h and 48 h or (B) D11 for 24 h. Control cells (CT) were treated with 0.1% DMSO. The expression of the indicated proteins was verified by Western blot analysis. β-actin was detected as loading control. (C) Phase-contrast microscopy pictures of U-87 MG cells treated with vehicle (24 h), 0.5 μM 17-AAG (24 h), 50 μM D11 (5 h or 24 h) or the combination. Combination (1) refers to cells treated with 17-AAG and D11 for 24 h. Combination (2) refers to cells treated with 17-AAG for 24 h and D11 added in the last 5 h of incubation time. Cell pictures were taken at 100x magnification. (D) U-87 MG cells were treated as described in (C). Expression of the indicated proteins was analyzed by Western blot. (E) and (F), M059K cells were treated and analyzed essentially as described in (C) for 24 h. Experiments were performed three times obtaining similar results.

We initially analyzed the levels of expression of HSP90 in glioblastoma cells treated with 17-AAG choosing a range of concentrations of the compound based on results from preliminary experiments (S1A Fig). Cell incubation with up to 0.5 μM 17-AAG for 24 and 48 h, respectively, resulted in inhibition of HSP90 chaperone activity as indicated by the reduced expression levels of EGFR a client protein whose stability is dependent on HSP90 functional interaction (Fig 1A, [24]). The expression of HSP90 was unchanged, however, we observed induction of HSP70 expression in a time and concentration-dependent manner. We, then, analyzed effects of CK2 inhibition on the endogenously expressed chaperone proteins employing the recently identified small-molecule inhibitor D11 [21,25]. Cell treatment with increasing concentrations of D11 resulted in increased cytotoxicity (S1B Fig) and, concomitantly, inhibition of CK2 activity as demonstrated by decreased phosphorylation of NF-κB/p65 at S529 a known CK2 target site (Fig 1B, [26]), however, it did not result in a significant change in the levels of expression of HSP90 and HSP70. Next, we investigated whether D11 affected 17-AAG-induced HSP70 expression levels. Combination treatment resulted in morphological changes such as rounding up and partial loss of adhesion (Fig 1C), which were more pronounced following 24 h incubation time as compared to control experiments. Analysis of whole cell lysates by Western blot revealed that treatment with D11 caused inhibition of endogenous CK2 activity as indicated by the levels of phosphorylation of NF-κB at S529. Interestingly, combination treatment was accompanied by a significant drop in HSP70 expression most prominent at 24 h incubation time as compared to cells treated with 17-AAG alone (Fig 1D). To verify that this effect was not limited to one glioblastoma cell line, experiments were also carried out employing M059K cells obtaining similar results (Fig 1E and 1F).

**Fig 2:**
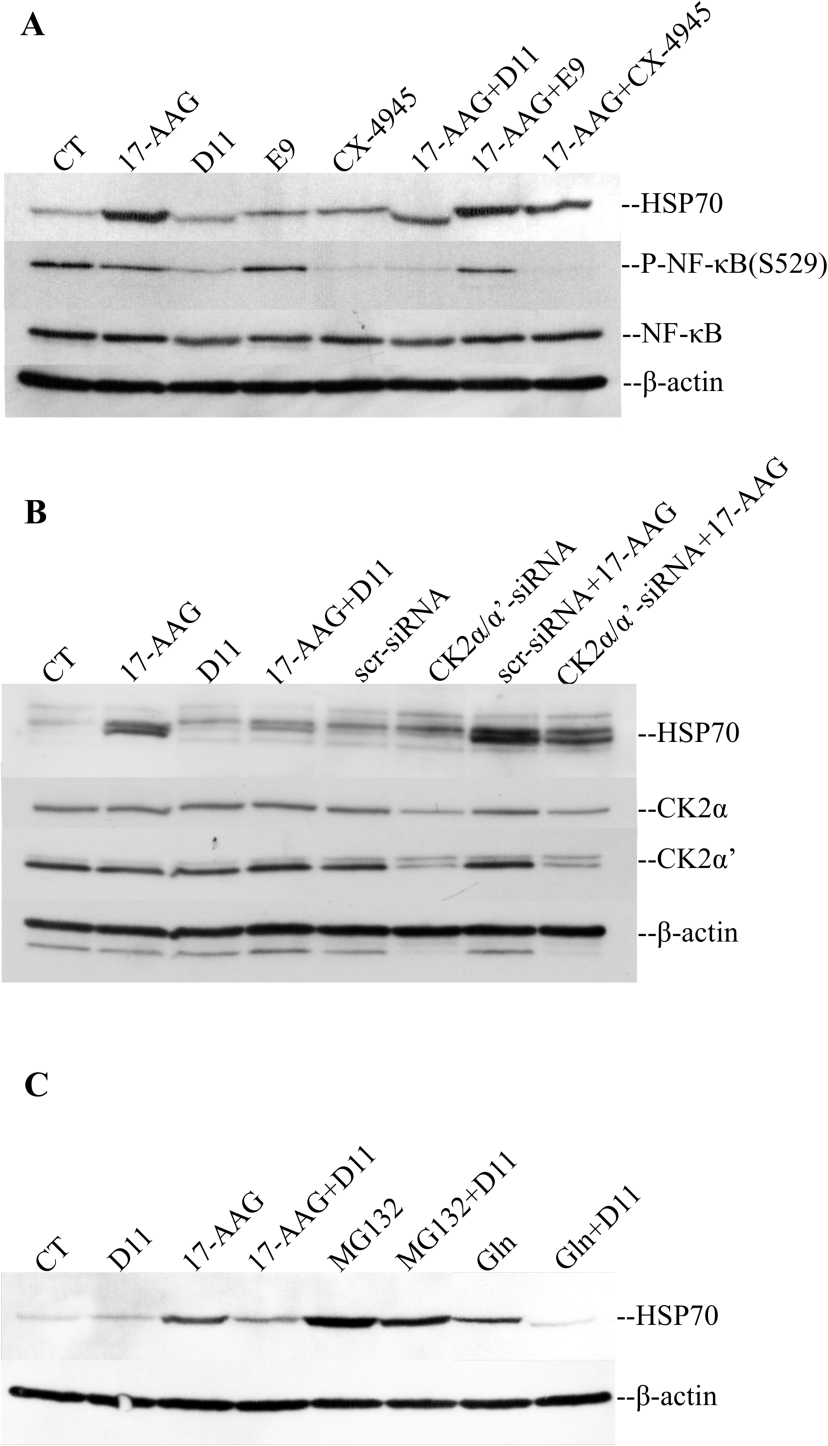
Inhibition of protein kinase CK2 leads to destabilization of HSP70. (A) U-87 MG cells were treated with 0.5 μM 17-AAG in combination with 50 μM D11, 50 μM E9 and 20 μM CX-4945, respectively, for 24 h. Control experiments included cells incubated with 0.1% DMSO (CT) and the individual compounds at concentrations indicated above, respectively. (B) Cells were incubated with 50 μM D11 for 24 h or transfected with siRNA against the individual catalytic subunits of CK2 for 72 h. Control cells were either incubated with 0.1% DMSO for 24 h or transfected with scramble siRNA (scr-siRNA) for 72 h. Where indicated, cells were additionally incubated with 0.5 μM 17-AAG for 24 h before harvesting. (C) Cells were treated with 0.5 μM 17-AAG, 50 μM D11, 50 μM MG132 and 2 mM glutamine (Gln), respectively. Cells were treated with 17-AAG and/or D11 for 24 h while MG132 and Gln were added 12 h before harvesting, respectively. The expression of proteins indicated in the figure was analyzed by Western blot. Experiments were performed three times obtaining similar results. β-actin detection served as a control for equal loading.

For the majority of the following experiments U-87 MG cells were chosen, which are a widely accepted model in brain cancer studies. To investigate the particular involvement of CK2, destabilization of HSP70 was further tested with two additional CK2 inhibitors, i.e. E9 and CX-4945 [13,27]. Combination of D11 and 17-AAG was the most effective treatment in reducing the expression of HSP70 to roughly 50% with respect to cells treated with 17-AAG alone (Fig 2A). Next, we tested effects of CK2 down-regulation on HSP70 stability. Cells were transfected with scr-siRNA or siRNA directed against the individual catalytic subunits of CK2. As shown in Fig 2B, down-regulation of CK2 resulted in decreased levels of expression of HSP70 although not to the same extent as observed with D11 treatment suggesting that D11 regulates the expression of HSP70 through mechanisms that are partially dependent on intact CK2 function.

Next, to evaluate whether D11 was able to suppress HSP70 induction in the presence of other stressors we treated cells with the proteasome inhibitor MG132 and glutamine, respectively. As shown in Fig 2C, MG132 treatment was the most effective inducer of HSP70 up-regulation followed by 17-AAG and glutamine. Conversely, combined treatment with D11 resulted in lower expression levels of HSP70 as compared to control experiments.

### Cell treatment with D11 leads to inhibition of heat shock factor 1 (HSF1) transcriptional activity

**Fig 3:**
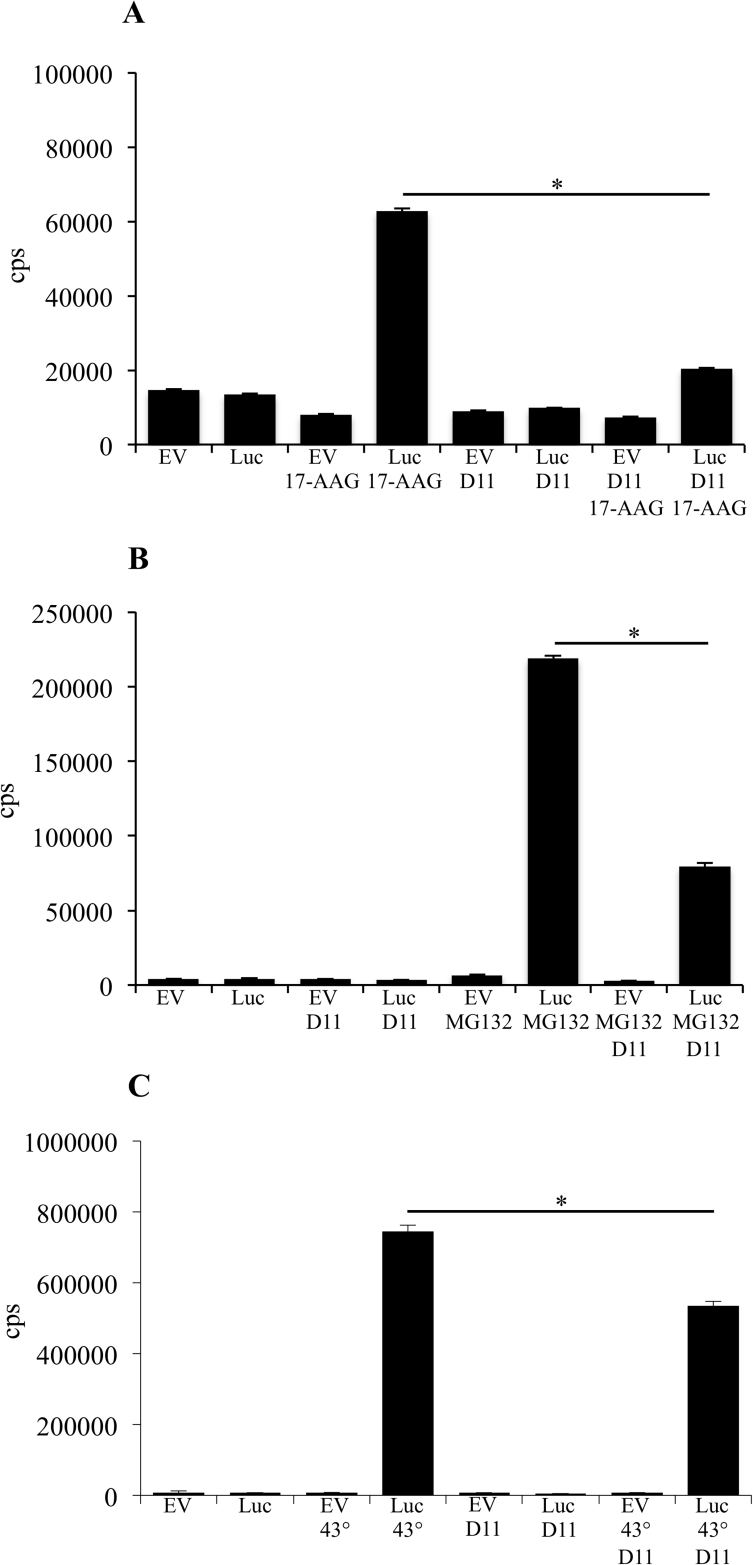
Inhibition of CK2 blocks HSF1 transcriptional activity. (A) Cells were transfected with a control vector (EV) or a vector carrying a HSF1 response element (Luc). After 24 h from transfection, cells were treated with 0.1% DMSO, 0.5 μM 17-AAG and 50 μM D11 for additional 24 h, respectively, as indicated in the figure. HSF1 transcription activity was measured by a luciferase reporter assay and 10 μg whole cell lysate. (B) Experiments were carried out as in (A). Where indicated, cells were incubated with 50 μM MG132 in the last 12 h of incubation time. (C) Experiments were conducted as described in (A). After 24 h from transfection, cells were incubated at 43°C for 1 h and additional 23 h at 37°C before harvesting. Experiments were repeated twice in quadruplicates obtaining similar results. HSF1 transcription activity is expressed in counts/s (cps). Mean values +/- STDEV, *P < 0.0001.

The binding of 17-AAG to HSP90 results in de-repression of HSF1 activity leading to induction of the heat shock response [28]. Hence, we analyzed whether D11 affected the transcriptional activity of HSF1 in the presence of three different stressors by performing a luciferase reporter assay. Incubation of cells at 43°C for 1 h resulted in the highest level of activation of HSF1 (745,661 cps, Fig 3C) followed by MG132 (219,162 cps, Fig 3B) and 17-AAG (62,717 cps, Fig 3A). In contrast, co-treatment with D11 resulted in significantly lower luciferase expression in all cases suggesting that D11 induced down-regulation of HSP70 by inhibiting HSF1 transcriptional activity.

**Fig 4:**
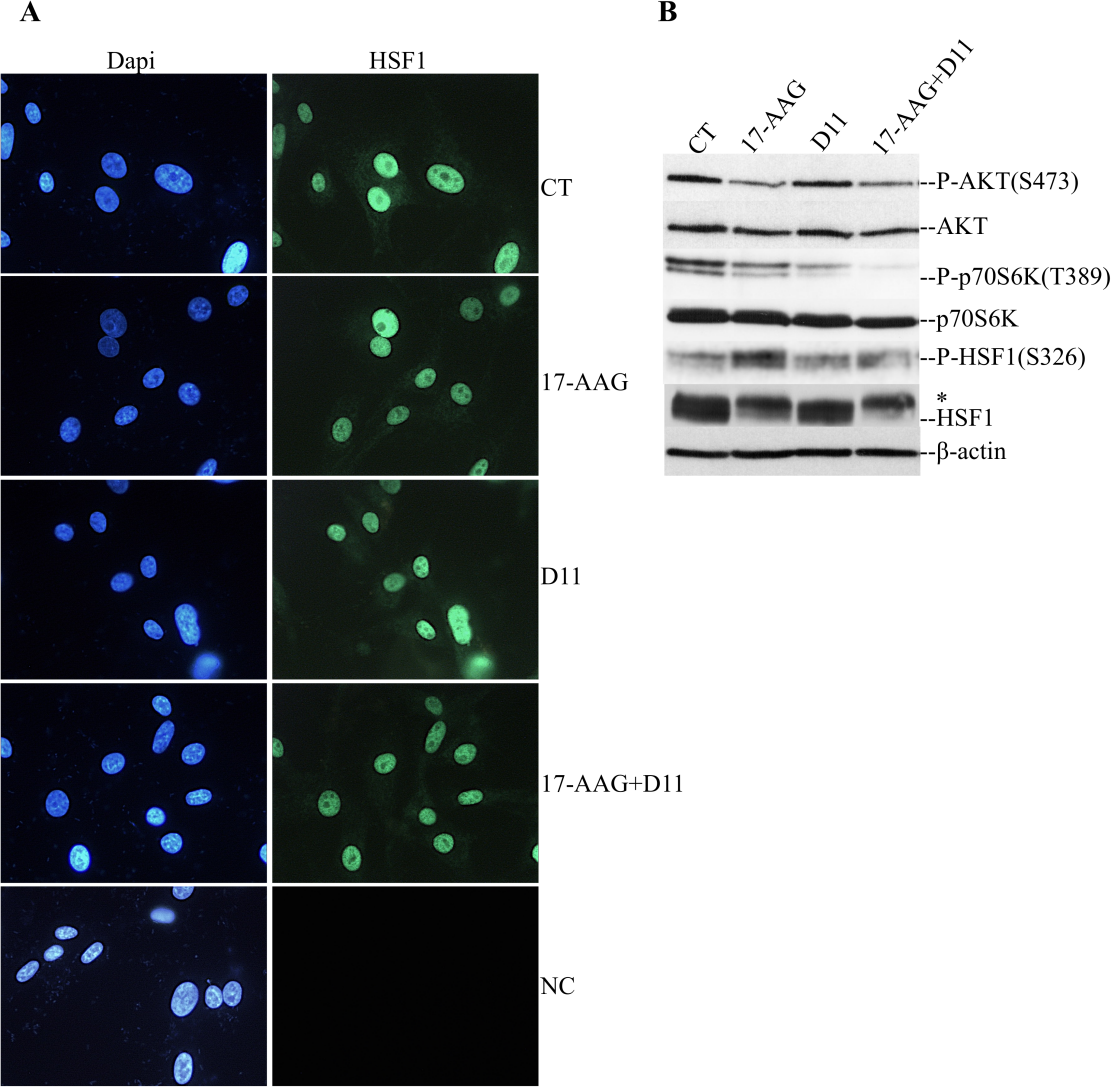
Cell treatment with D11 leads to dephosphorylation of HSF1 at S326. (A) Fluorescence-based pictures of cells treated with 0.5 μM 17-AAG, 50 μM D11 or the combination for 24 h. Control cells were treated with 0.1% DMSO for 24 h. Cells were labeled with anti-HSF1 antibody and biotin-conjugated secondary antibody. It followed staining with Alexa Fluor 488-conjugated streptavidin. Cell nuclei were visualized with DAPI staining. Cell pictures were taken at 400X magnification. NC: negative control. (B) Cells were treated as described above. Whole cell lysates were analyzed by Western blot employing antibodies against proteins indicated in the figure. The asterisk indicates hyperphosphorylated HSF1. Experiments were repeated three times obtaining similar results.

It is hypothesized that conversion of HSF1 to its active form is a multi-step process involving possible translocation to the nucleus and acquisition of DNA binding ability [29,30]. In order to analyze the subcellular localization of HSF1 we used immunofluorescent labeling of cells under control conditions as well as after treatment as indicated in Fig 4A. HSF1 was found predominantly localized in the nucleus in all samples and only a faint signal was visible in the cytoplasm. These results are entirely consistent with previously published data [30] and support the notion that nuclear translocation is not part of the multi-step process of activation of HSF1.

HSF1 is post-translationally modified by phosphorylation and sumoylation and these modifications are thought to modulate the activity of HSF1. Some of these modifications are inhibitory for transcription (i.e. S303, S307 and S308) while others appear to be important for the induction of HSF1 activity (i.e. S230, S320, S326, T142 and S419, [29,31]). In this respect, phosphorylation of HSF1 at S326 has been shown to be a critical modification following stress-induced HSF1 activation, specifically mediated by mammalian target of rapamycin in complex with raptor (i.e. TORC1 complex) and not rictor (i.e. TORC2 complex) and required for the heat- induced activation of the *HSP70*.*1* promoter [28]. In our hands, phosphorylation of HSF1 at S326 was promptly detected in cells treated with 17-AAG for 24 h as compared to control experiment (Fig 4B). However, simultaneous treatment with D11 resulted in signal intensity comparable to the one observed in control and D11-treated cells, respectively. Interestingly, upon activation by 17-AAG, HSF1 became phosphorylated and this could indirectly be seen by a slower migration of the protein on SDS-PAGE as also previously described [32,33]. Simultaneous treatment with D11 resulted in decreased phosphorylation of HSF1 at S326, however, HSF1 protein appeared to migrate slightly more slowly as compared to treatment with 17-AAG. Owing to the fact that cell treatment with D11 leads to inhibition of HSF1 transcription activity (Fig 3), results reported in Fig 4B suggest that combination treatment might induce a phosphorylation state that is inhibitory and independent from the phosphorylation status at S326. In order to confirm that decreased phosphorylation of HSF1 at S326 resulted from inhibition of the TORC1-mediated pathway, we analyzed the phosphorylation levels of p70S6K at T389 and AKT at S473 that are specific downstream targets of TORC1 and TORC2 complexes, respectively [34]. Results presented in Fig 4B show that in U-87 MG cells, mTOR in complex with raptor is mostly affected by treatment with D11 alone or in combination with 17-AAG suggesting a preferential inhibition of TORC1 complex.

### Differential gene expression profile analysis of U-87 MG cells in response to 17-AAG treatment in combination with D11

**Fig 5:**
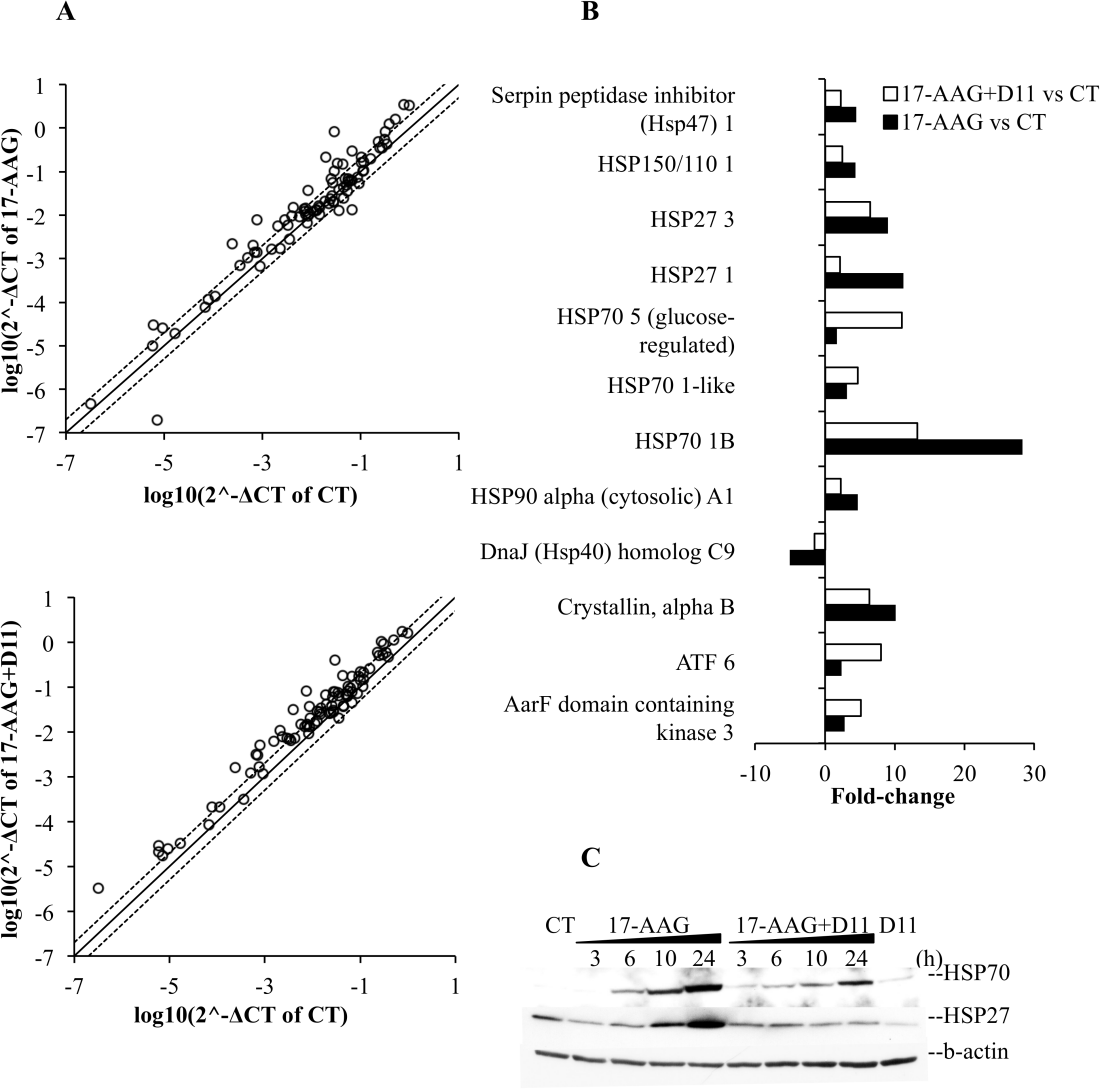
Differential gene expression profiling in glioblastoma cells following stress induction. U-87 MG cells were incubated with 0.5 μM 17-AAG alone or in combination with 50 μM D11 for 24 h. (A) Global gene expression changes between cells incubated with 0.1% DMSO and 17-AAG are shown in the upper scatter plot. Changes of gene expression between cells treated with vehicle and a combination of 17-AAG and D11, respectively, are indicated in the lower scatter plot. The fold regulation cut-off (dashed line) was set to 2. (B) Differential up- or down-regulation of genes of the HSP90/HSP70/HSP60/small HSPs group following treatment as indicated above. (C) Whole cell lysates from cells treated with 0.5 μM 17-AAG, 50 μM D11 or the combination for increasing amounts of time were analyzed by Western blot employing antibodies directed against the indicated proteins. Signal from β-actin detection was used as loading control. Experiments were repeated two times obtaining similar results.

In order to establish differential mRNA expression in glioblastoma cells resulting from treatment of cells with 17-AAG alone or in combination with D11, we examined the expression of 84 heat shock protein genes that regulate protein folding using the human heat shock proteins and chaperones RT^2^ profiler PCR array (Fig 5A). The log of the fold-change in gene expression of 17-AAG-treated cells was plotted against log of the fold-change in gene expression of control cells (Fig 5A, upper plot) while log of the fold-change in gene expression of combination-treated cells was plotted against log of the fold-change in gene expression of control cells (Fig 5A, lower plot). The expression of several genes was unchanged after treatment with 17-AAG alone or in combination with D11, as compared to control experiments, while the fold-change of gene expression especially for genes belonging to the HSP40 family was approximately 2 following combination treatment (Fig 5A and data not shown). However, this was not the case for seven of the genes that were analyzed. *HSP70 1B* expression was found up-regulated to a great extent after treatment with 17-AAG and the expression declined as a result of combination treatment. A similar trend was observed in the case of six other heat shock proteins, namely, *Serpin peptidase inhibitor*, *HSP150/110 1*, *HSP27 1*, *HSP27 3*, *HSP90*α *A1* and *Crystallin*, *alpha B*. Conversely, combination treatment resulted in the up-regulation mainly of *HSP70 5*, *ATF 6* and *AarF domain containing kinase 3* (Fig 5A and 5B).

Similarly to HSP70 and HSP90, the small chaperone HSP27 increases to become one of the dominantly expressed proteins in stress conditions (reviewed in [35]). Moreover, HSP27 is highly expressed in high-grade astrocytoma [36]. Hence, we investigated whether down-regulation of *HSP27* was accompanied by changes in the expression levels of the corresponding protein (Fig 5C). Treatment with 17-AAG for up to 24 h resulted in increased expression of HSP27. Conversely, simultaneous treatment with D11 led to a correspondent decrease in protein signal for up to 24 h incubation.

Overall, the observed differences in gene expression suggest that treatment of glioblastoma cells with D11 results in the negative modulation of a restricted subset of genes linked to cellular stress and involved in resistance to radio- and chemotherapy.

### Simultaneous treatment with 17-AAG and D11 results in significant cell toxicity

**Fig 6:**
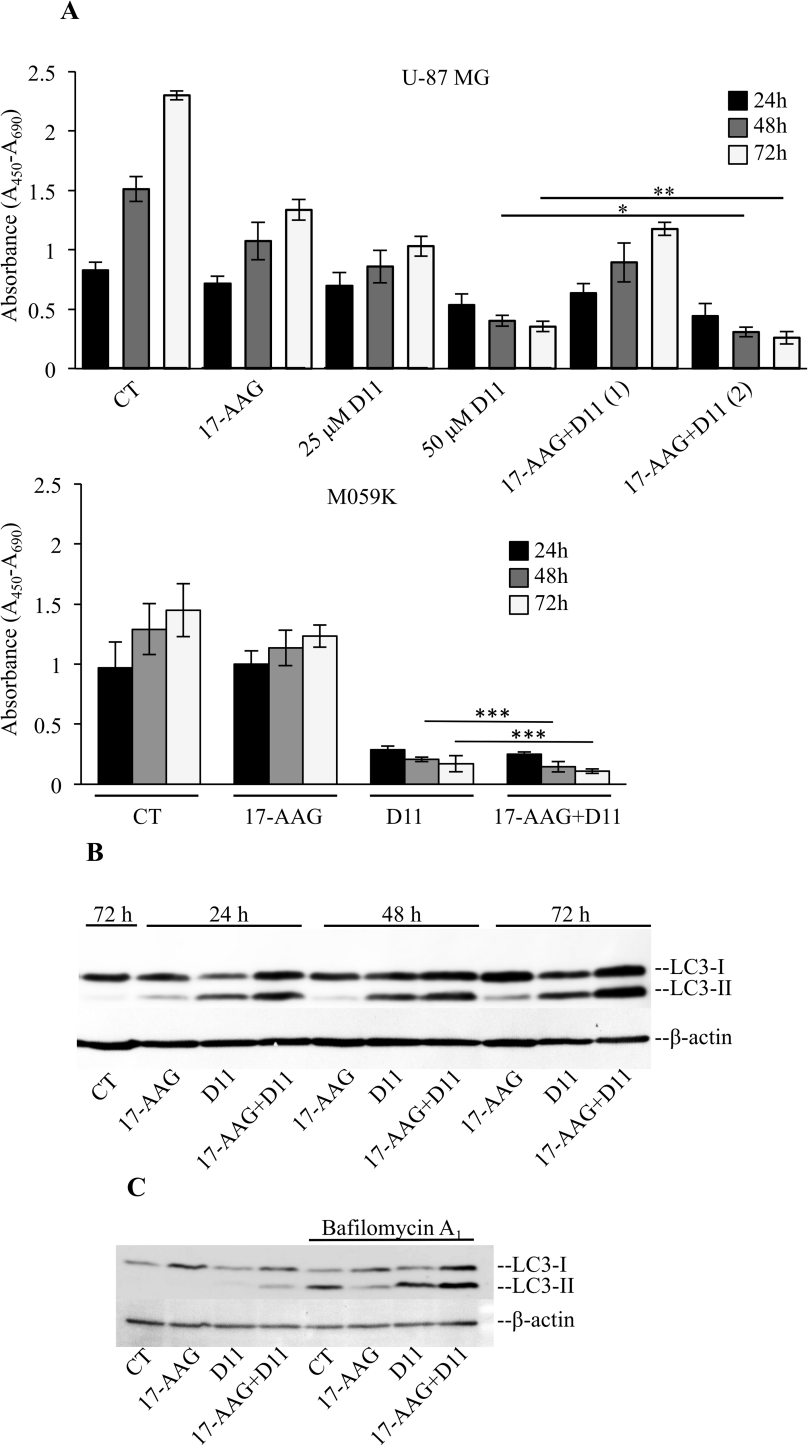
Simultaneous treatment with 17-AAG and D11 leads to cytotoxicity and activation of autophagy in a time-dependent manner. (A) Cells were treated with 0.5 μM 17-AAG alone or in combination with 25 μM D11 (Combination 1) and 50 μM D11 (Combination 2), respectively, for the indicated times. Control experiment (CT) refers to cells treated with 0.1% DMSO. The proportion of viable cells was determined by WST-1 assay and expressed in arbitrary units as a difference in absorbance measured at 450 nm and 690 nm (reference) wavelengths, respectively, (mean values +/- STDEV, *N = 6*). Asterisks denote statistically significant differences between the indicated sets of data, *P < 0.005, **P < 0.01, ***P < 0.05. (B) U-87 MG cells were incubated with 0.5 μM 17-AAG and 50 μM D11 as indicated in the figure for increasing amounts of time. Western blot analysis of whole cell lysates employing antibodies against the indicated proteins is shown. (C) LC3-I and -II expression levels were determined after incubation of U-87 MG cells with 17-AAG and D11 at concentrations as in (A) for 24 h. Where indicated, 100 nM Bafilomycin A_1_ was added in the last 6 h of incubation time. Experiments were performed three times obtaining similar results.

In order to determine effects on cell viability, we treated cells with 17-AAG alone or in combination with D11, respectively, as indicated in Fig 6A. Incubation with 17-AAG alone partially affected cell viability as compared to control experiments. Conversely, treatment with 50 μM D11 resulted in loss of cell viability. However, combination treatment led to a further statistically significant reduction in the number of viable cells assessed at 24 h to 72 h. Based on results reported above, we asked whether combination treatment stimulated autophagy. In this respect, it has been shown that HSF-1 inhibits autophagy *via* one of its principal effectors, HSP70, establishing a direct link between the heat shock response and autophagy system [37–39]. Western blot analysis of whole cell lysates showed induction of autophagy in cells treated with D11 in a time-dependent fashion as supported by the conversion of light chain 3 (LC3-I) to LC3-II. Conversely, treatment with 17-AAG alone appeared to slightly increase LC3-II levels. However, a significant increase in LC3-II levels occurred when cells were incubated with 17-AAG and D11 for up to 72 h (Fig 6B). In order to determine whether high LC3-II levels resulted from increased generation of autophagosomes or a decrease in the rate of autophagosome clearance, cells were incubated with bafilomycin A_1_ a lysosomal proton pump inhibitor [40]. As shown in Fig 6C, combination treatment induced LC3-II levels to rise, however, additional treatment with bafilomycin A_1_ resulted in a further increase in LC3-II signal intensity indicating that combination treatment leads to induction of autophagic flux.

Taken together, these data indicate that incubation of glioblastoma cells with HSP90 inhibitors in combination with D11 promotes down-regulation of HSP70 and, concomitantly, induction of autophagy in a time-dependent manner.

## Discussion

Compelling evidence has indicated that although inhibitors of HSP90 disrupt the chaperone machinery, they often lead to increased expression of other heat shock proteins neutralizing the beneficial effects deriving from HSP90 inhibition. In this respect, previously published data and own preliminary results indicated that reduction of the expression of HSP70 and/or HSC70 by siRNA interference and at the same time incubation with HSP90 inhibitors lead to increased toxicity in cancer cells ([41], S2 Fig). By the same token, Holmes *et al*., [42] showed that knockdown of AHA1 resulted in a significant increase in sensitivity to 17-AAG resulting from decreased phosphorylation of key cellular proteins such as c-RAF, in a panel of human cancer cell lines. However, although such a strategy appears to be very promising, one should not underestimate the difficulties to induce down-regulation of protein expression in patients’ cells.

In our study, we show for the first time that simultaneous treatment of glioblastoma cells with 17-AAG and D11 leads to significant decrease of HSP70 and HSP27 expression levels. We showed that the ability of D11 to counteract induction of HSP70 applies to different types of stressors. However, D11 effects seem to be partially dependent on CK2 inhibition. In this respect, treatment of cells with CX-4945, another potent and selective inhibitor of CK2, resulted in only a slight decrease of HSP70 expression levels while in the case of D11 the drop was substantial despite the fact that both compounds could efficiently inhibit the kinase activity of endogenous CK2 (Fig 2A).

The expression of heat shock proteins is dependent on activation of a family of heat shock transcription factors of which HSF1 is the best characterized and essential for the heat shock response. It has been shown that the level of activation of HSF1 depends on the type of stress stimulus and heat stress appears to be the most effective one [32]. Accordingly, we could confirm this observation but also demonstrate that cell incubation with D11 leads to inhibition of HSF1 transcription activity. While decreased expression of HSP70 and HSP27 might derive from inhibition of HSF1 transcriptional activity, one cannot exclude that lack of phosphorylation of the co-chaperone CDC37, which is a target of CK2 and binding partner of HSP90 [43], might contribute to lack of stabilization of pro-survival chaperones (e.g. HSP70, HSP27). In this respect, accumulation of HSP70 following apoptosis induction has been shown to occur independently of increased gene expression and rather through a post-translational modification mechanism [44].

Phosphorylation of HSF1 at S326 was identified as a modification that is critical to stress-induced HSF1 activation [45]. This post-translational modification was shown to be catalyzed by mTOR, however, recent studies have revealed that the MAPK-mediated signaling might also contribute to the phosphorylation of S326 following incubation with the dietary agent phenethyl isothiocyanate [46]. In our hands, we could not detect any appreciable modification in the kinase activity of p38 MAPK in cells incubated with 17-AAG, D11 or with a combination of both compounds (results not shown). The fact that phosphorylation of HSF1 at S326 was found reduced in cells treated with 17-AAG and D11 and that was accompanied by inhibition of TORC1 activity, suggests that activation of HSF1 through phosphorylation at S326 might be stimulus- or even cell-type specific.

The gene expression profiling showed up-regulation of *HSP70 5*, *ATF* and *AarF domain containing kinase 3* following combination treatment.

*HSP70 5*, also known as GRP78, is a HSP70-type molecular chaperone of the endoplasmic reticulum (ER) up-regulated under stress conditions *via* the unfolded protein response (UPR, reviewed in [47]). Although it cannot be excluded that up-regulation of *HSP70 5* expression derives from increased levels of unfolded/missfolded proteins as a result of attenuated HSP90/CDC37 complex formation, it cannot be excluded that enhanced expression of *HSP70 5* results from autophagy induction which is seen in combination treatment. Consistent with this notion, studies conducted with human cell lines have shown that HSP70 5 regulates ER stress-induced autophagy by stimulating autophagosome formation [48].

It has been suggested that increase in autophagic markers and autophagic flux should be simultaneously detected in order to support a direct role of autophagy in cell death [49]. Simultaneous treatment with 17-AAG and D11 resulted in decreased viability and a dramatic increase in autophagy induction supporting the involvement of this process in the observed cellular toxicity. At any rate, given the ongoing controversy as to which extent autophagy exerts a cytoprotective role or represents a separate form of cell death, one should consider that even as a form of cell defense, persistent stimulation of autophagy can lead to a caspase-dependent or -independent form of cell death [49].

Various HSP90 inhibitors have entered clinical trials based on the notion that these synthetic inhibitors are more potent in cancer cells than in normal cells and that inhibition of HSP90 would destabilize an extensive number of oncoproteins de-regulated in cancer cells. However, as mentioned above, rapid induction of members of the heat shock protein family including HSP70 is often observed resulting in attenuation of the cytotoxic effects deriving from HSP90 inhibition.

The usage of structurally unrelated HSP90 inhibitors that lack quinone moiety has been suggested in order to overcome resistance to 17-AAG treatment as it often results in low levels of NAD(P)H/quinone oxidoreductase (NQO1). This enzyme reduces the quinone moiety of 17-AAG to its dihydroquinone which has higher HSP90 binding properties [10,50]. However, one should emphasize the complexity of the HSP90-mediated response, which involves post-translational modifications, interaction with co-chaperones and regulation of client proteins stability underlining the fact that only a careful analysis of the tumor biology and the environmental context in which the tumor is thriving may determine the choice of optimal HSP90-targeted therapeutics.

In this study, we show that combined treatment of 17-AAG with D11 leads to enhanced cytotoxicity and is accompanied by significant decrease in HSP70 and HSP27 expression levels suggesting that 17-AAG-mediated induction of the stress response may actually be required for glioblastoma cells to tolerate damage induced by chaperone protein inhibitors.

Overall, data reported here suggest that co-treatment of glioblastoma cells with inhibitors of HSP90 and compounds that compromise the stress response represents a promising therapeutic strategy that warrants further consideration particularly in the case of acquired resistance towards single chemotherapeutic agents targeting HSP90.

## Supporting information

S1 Fig Dose response to 17-AAG and D11, respectively, determined by cell viability assay. U-87 MG cells were treated with increasing concentrations of 17-AAG (A) or D11 (B) for 24 h, 48 h and 72 h, respectively. Cell viability was determined as indicated in Fig 6A. Control experiments refer to cells incubated with 0.1% DMSO. IC_50_ values are shown in the inserts (mean values +/- STDEV, *N = 6*).
(TIF)

S2 Fig siRNA-mediated silencing of HSP70 increases sensitivity of glioblastoma cells to 17-AAG treatment. U-87 MG cells were transfected with scramble (scr) or siRNA directed against HSP70 for 72 h. 24 h before the end of the experiments, cells were incubated with 0.1% DMSO or 17-AAG as indicated in the figure. Cell viability expressed in percentage was essentially determined as described in Fig 6A. The bar graph shows mean values +/- STDEV, *N = 12*; *P < 0.05.
(TIF)
