# Supplementary figures and images for: The small-molecule kinase inhibitor D11 counteracts 17-AAG-mediated up-regulation of HSP70 in brain cancer cells
Source: PLoS One. 2017 May 18;12(5):e0177706. doi: 10.1371/journal.pone.0177706 (PMC5436671; doi:10.1371/journal.pone.0177706)

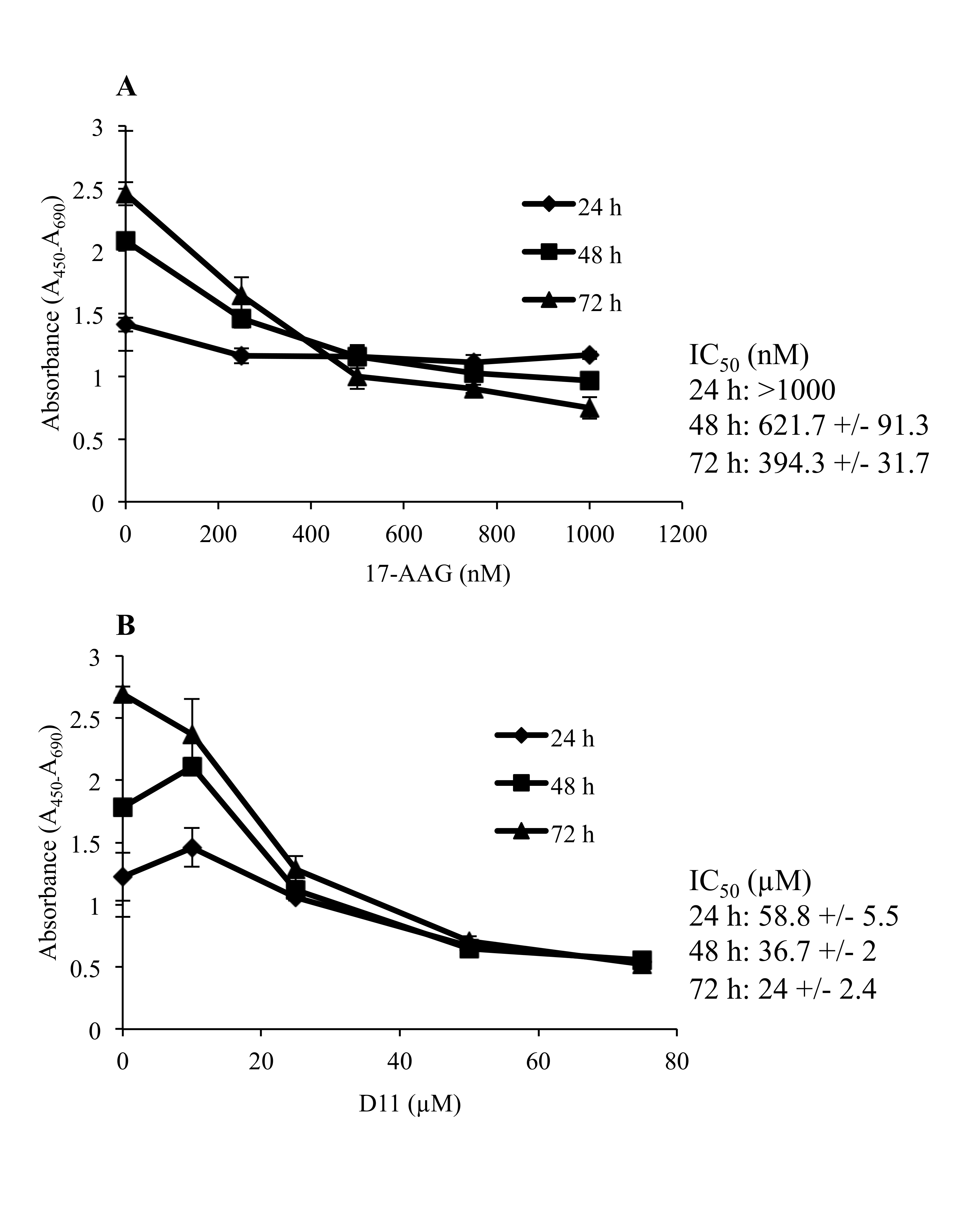

Supplement: S1 Fig — U-87 MG cells were treated with increasing concentrations of 17-AAG (A) or D11 (B) for 24 h, 48 h and 72 h, respectively. Cell viability was determined as indicated in Fig 6A. Control experiments refer to cells incubated with 0.1% DMSO. IC50 values are shown in the inserts (mean values +/- STDEV, N = 6). (TIF) [file pone.0177706.s001.tif]

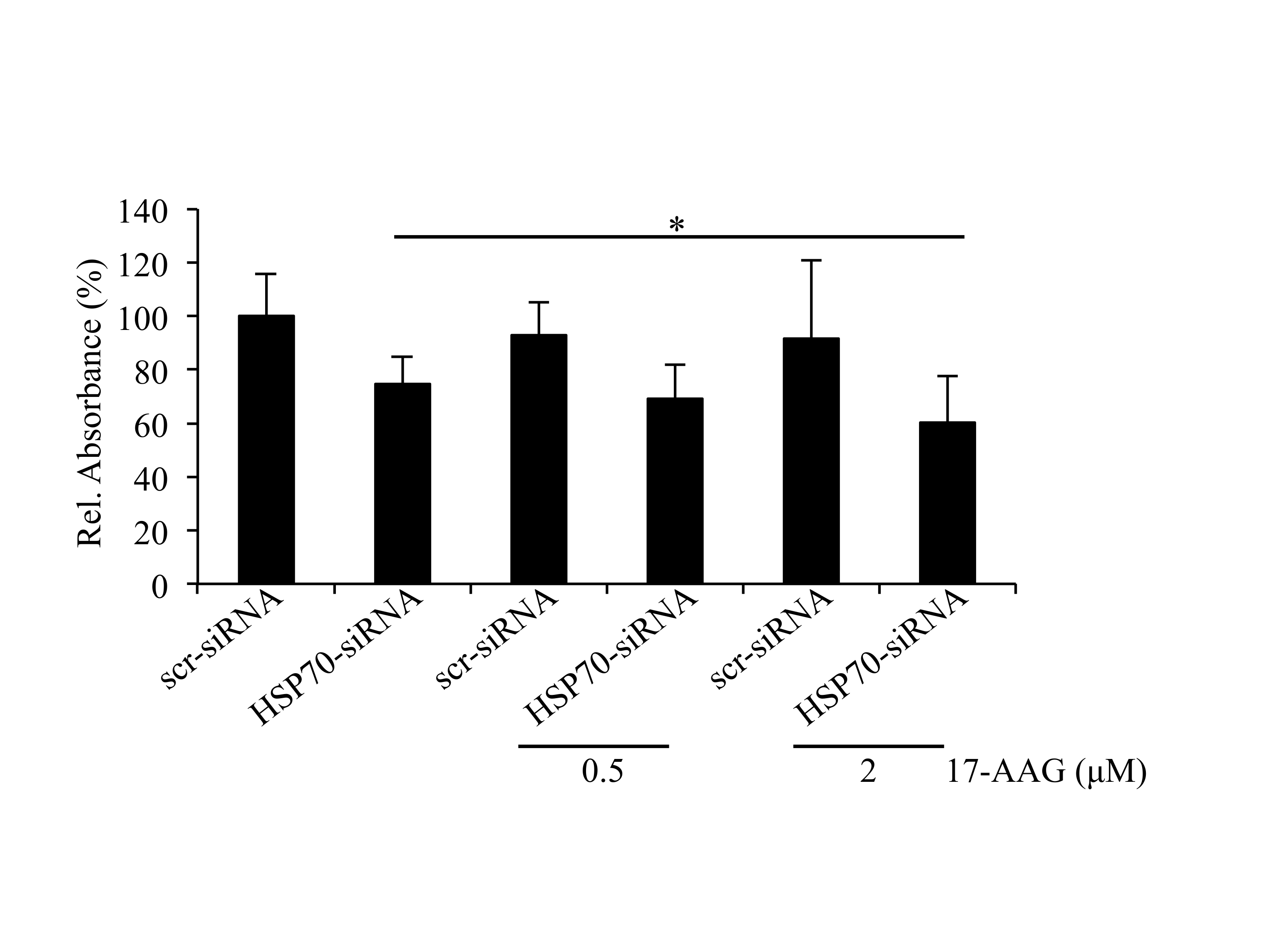

Supplement: S2 Fig — U-87 MG cells were transfected with scramble (scr) or siRNA directed against HSP70 for 72 h. 24 h before the end of the experiments, cells were incubated with 0.1% DMSO or 17-AAG as indicated in the figure. Cell viability expressed in percentage was essentially determined as described in Fig 6A. The bar graph shows mean values +/- STDEV, N = 12; *P < 0.05. (TIF) [file pone.0177706.s002.tif]
